# Supplementary material for: On the analysis of functional PET (fPET)-FDG: Baseline mischaracterization can introduce artifactual metabolic (de)activations
Source: Imaging Neurosci (Camb). 2025 Aug 28;3:IMAG.a.110. doi: 10.1162/IMAG.a.110 (PMC12395282; doi:10.1162/IMAG.a.110)
Supplement: Supplementary Material [file IMAG.a.110_supp.pdf]

Supplementary Materials

On the analysis of functional PET (fPET)-FDG: baseline mischaracterization can introduce artifactual metabolic (de)activations

Sean E. Coursey[a,b,*], Joseph Mandeville[a,c], Murray B. Reed[d,e], Grant A. Hartung[a,c], Arun Garimella[a], Hasan Sari[f], Rupert Lanzenberger[d,e], Julie C. Price[a,c], Jonathan R. Polimeni[a,c,g], Douglas N. Greve[a,c], Andreas Hahn[d,e], and Jingyuan E. Chen[a,c,*]

[a] Athinoula A. Martinos Center for Biomedical Imaging, Massachusetts General Hospital, Boston, MA, USA
[b] College of Science, Northeastern University, Boston, MA, USA
[c] Department of Radiology, Harvard Medical School, Boston, MA, USA
[d] Department of Psychiatry and Psychotherapy, Medical University of Vienna, Austria
[e] Comprehensive Center for Clinical Neurosciences and Mental Health (C3NMH), Medical University of Vienna, Austria
[f] Advanced Clinical Imaging Technology, Siemens Healthcare AG, Lausanne, Switzerland
[g] Harvard-MIT Program in Health Sciences and Technology, Massachusetts Institute of Technology, Cambridge, MA, USA

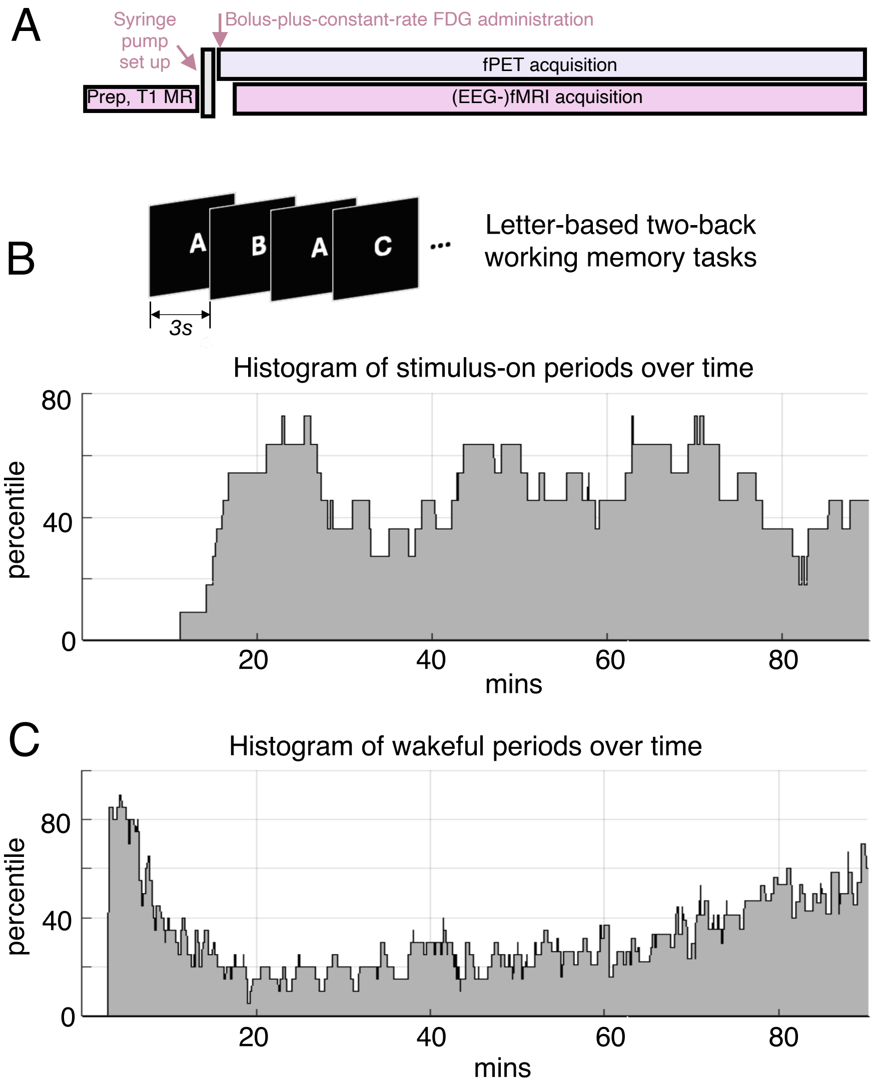

Figure S1: Summary of the fPET-fMRI experimental schemes of the bolus-plus-constant-infusion fPET-FDG dataset, collected at MGH. (A) Overview of the simultaneous fPET-FDG and BOLD-fMRI acquisitions. For all experiments, (EEG-)fMRI scans started a few minutes after the onset of the PET acquisition, and persisted throughout the entire experiment without interruption. **(B)** Working-memory dataset: histogram summary of the timing of stimulus-on/off blocks across 11 participants. Stimulus-on blocks (10–15 minutes): participants were instructed to judge whether a currently-present letter was identical to the one presented two letters back; stimulus-off blocks (10–15 minutes): viewing a fixation cross displayed at the center of the screen. **(C)** Endogenous-arousal dataset: histogram summary of the wakeful periods over the course of the experiment, estimated across 21 subjects with simultaneous EEG or behavioral data. This dataset was a subset of those collected to investigate sleep-induced changes in cerebral glucose metabolism in a separate study. To enhance sleep pressure during the experiment, subjects were sleep-deprived the night before the experiment, with their sleep restricted to only 4 hours.

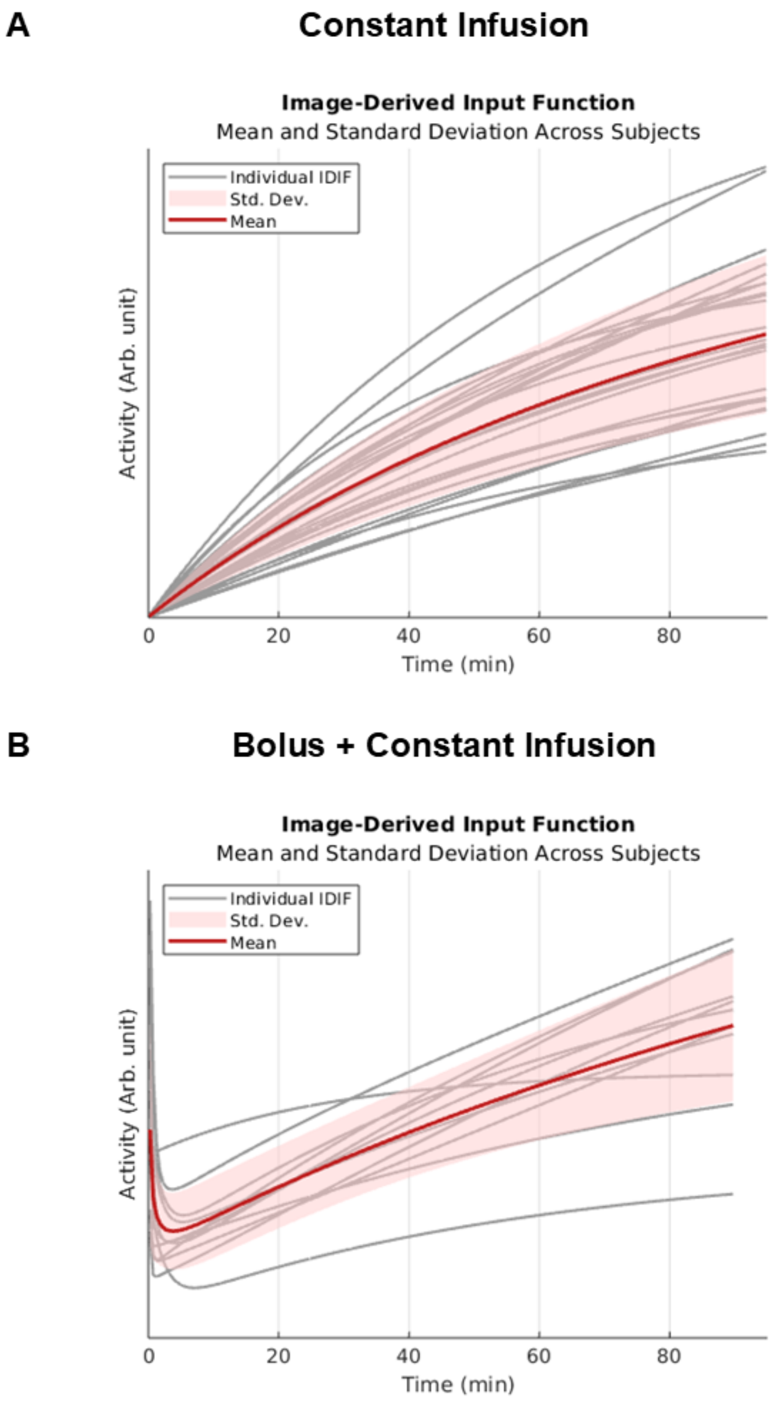

Figure S2: Mean and standard deviation of fitted image-derived arterial input functions across subjects. For each subject, Mean TACs were extracted from manually segmented carotid arteries with partial volume correction. These TACs were fitted by the convolution of two decaying exponentials with the infusion paradigm to remove noise. Finally, the mean and standard deviation across subjects was calculated. Since these image-derived input functions were used for their shape and not their absolute scale for our simulations, they are reported in arbitrary units.

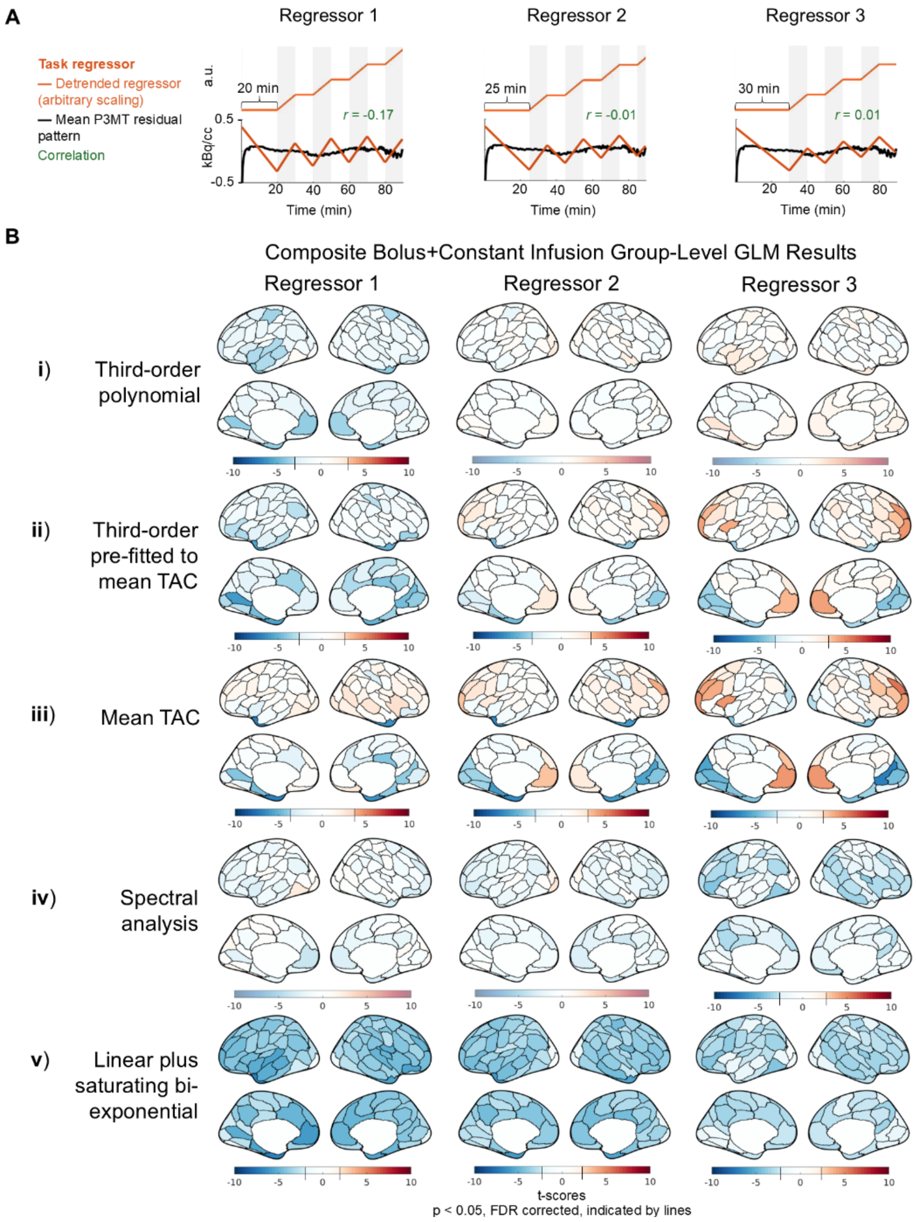

Figure S3: Summary of group-level random-effect t-scores from applying sham task regressors to the 100-parcel composite B+CI dataset with various baseline models, including the entire scan time for GLM analysis. The sham task regressors alternate between 10-minutes "on", modeled by a ramp with unit slope, and 10-minutes "off", modeled as flat. The sham task regressors differ in their initial rest period with Regressors 1, 2, and 3 having 20, 25, and 30 minutes of initial rest, respectively. The various baseline models comprise "Third-order polynomial (P3)", "Third-order pre-fitted to the mean TAC (P3MT)", "Mean TAC (MT)", "Spectral analysis (SA)", and "Linear plus bi-exponential model (EXP2)". To facilitate the visualization of artifactual metabolic (de)activations, the color bar uses a step-change in saturation at the significance threshold ($p < 0.05$, FDR), if such a threshold exists (Taylor et al., 2023). It should be

noted that the B+CI dataset, being a composite of working memory and endogenous arousal data, may exhibit more inter-subject variability than a typical study due to between-experiment differences, complicating interpretation. Additionally, the true task and arousal effects remain present at the single-subject level, although we expect jitter and randomness to mitigate these effects to insignificance at the group level. Different spatial patterns of artifactual activation are identified than in the CI dataset (Fig. 3) because the detrending methods interact differently with the shape of the B+CI baseline TAC as compared to the CI baseline TAC.

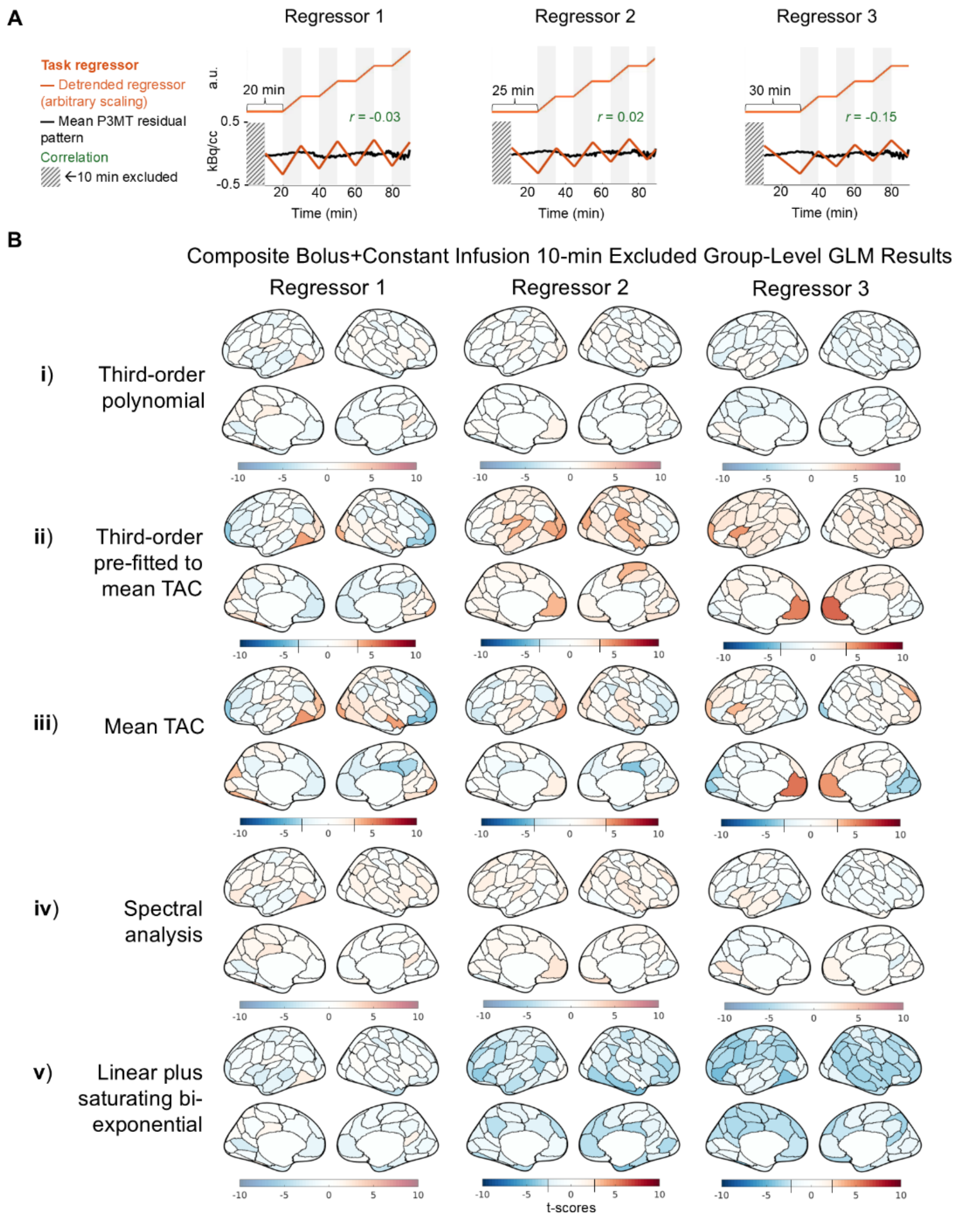

Figure S4: Summary of group-level t-scores from applying sham task regressors to the 100-parcel composite B+CI dataset with various baseline models, excluding the first 10 minutes the scan time for GLM analysis. Refer to the caption of Figure S4 for descriptions of task regressors, detrending methods, color scale schemes, and the B+CI dataset.

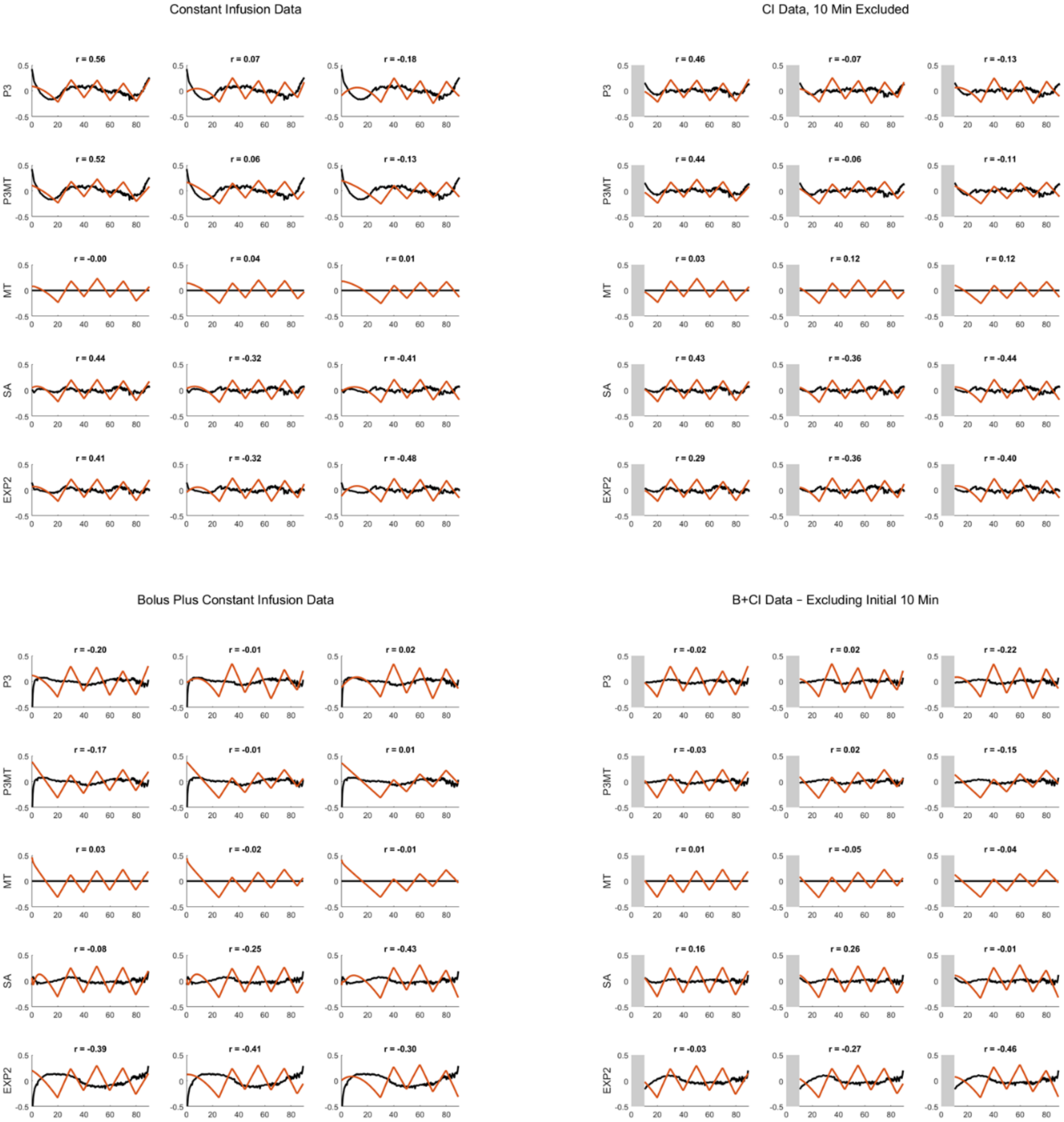

Figure S5: Comparison of mean residual patterns and mean detrended task regressors. For each dataset, constant infusion (top) and bolus plus constant infusion (bottom), including (left) and excluding (right) the first ten minutes, plots the mean residual pattern compared to the mean detrended task regressor across subjects for each detrending method / sham regressor combination. The detrending methods and sham regressors are those described in Fig. 3.

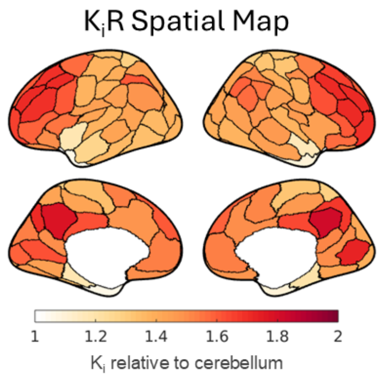

Correlation between |t-scores| and K_iR for CI data

Detrend Method	Regressor 1	Regressor 2	Regressor 3
P3	-0.41*	-0.46*	-0.03
P3MT	-0.65*	-0.02	-0.69*
MT	-0.20	-0.12	-0.04
SA	-0.36*	0.39*	-0.20
EXP2	-0.55*	0.37*	-0.18

Correlation between |t-scores| and K_iR for B+CI data

Detrend Method	Regressor 1	Regressor 2	Regressor 3
P3	-0.18	-0.23	-0.20
P3MT	-0.11	0.04	0.31*
MT	-0.28*	-0.07	0.16
SA	0.11	0.31*	0.38*
EXP2	-0.07	0.00	0.27*

* indicates $p < 0.01$ (uncorrected)

Figure S6: Spatial map of K_iR (left) and tables comparing spatial distribution of absolute t-scores to spatial distribution of K_iR (right). K_iR is the relative net influx rate compared to the cerebellum and represents the relative uptake of FDG, similarly to SUVR. K_iR values were calculated via Patlak plot analysis of the CI data and averaged across subjects; they are plotted using the same parcellation scheme as the results in Figs. 3, 4, S4, and S5 to facilitate comparison. The tables to the right list the Pearson correlation coefficients between the spatial distribution of K_iR and that of the absolute t-scores resulting from various regressor / detrending method combinations, for the CI (top) and the B+CI (bottom) datasets. The regressors and detrending methods are those from Figs. 3, 4, S2, and S3. We observe a range of significant and insignificant (anti)correlations, suggesting a complex relationship between regional FDG uptake and detrending-induced bias.

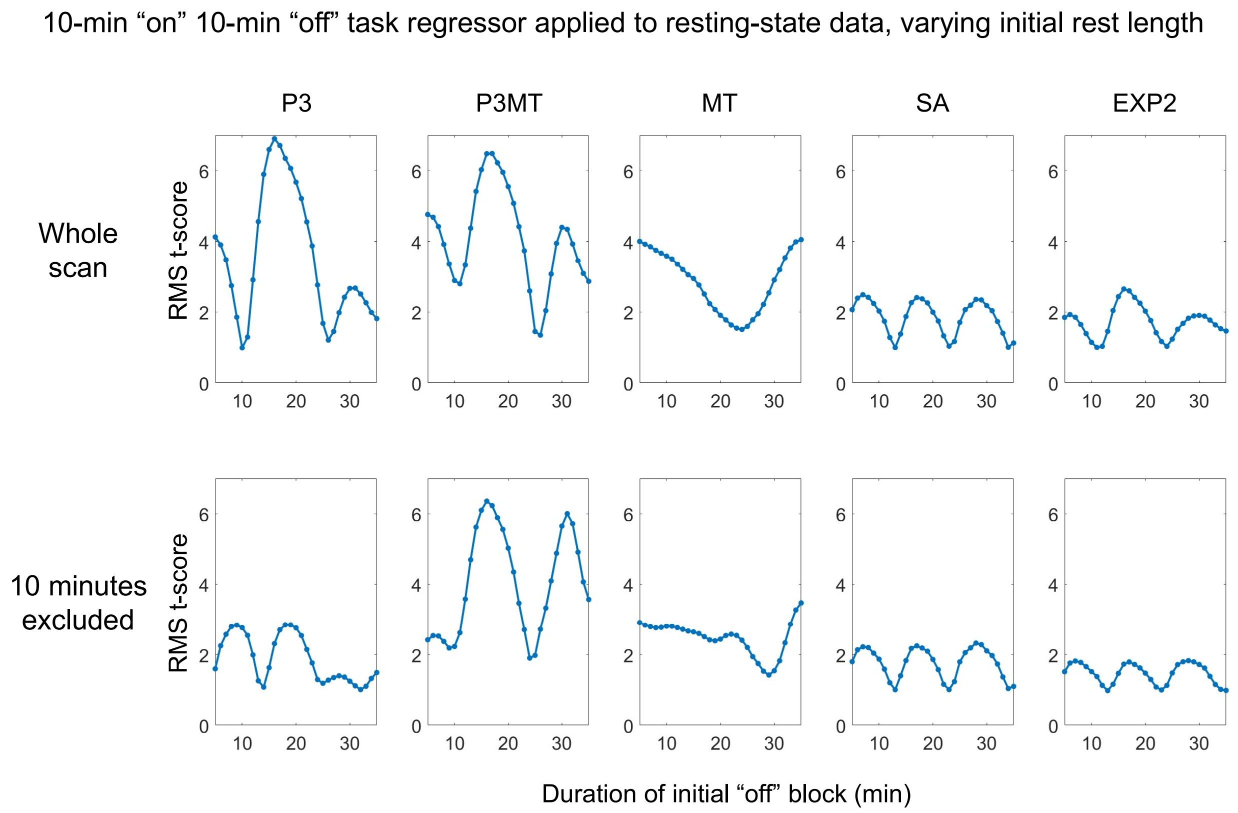

Figure S7: Summary of group-level random-effect root-mean-squared t-scores from applying a 10-min “on”, 10-min “off” faux task regressor with varying amounts of initial rest (from 5 to 35 minutes) to the 100-parcel resting-state dataset. Regressors 1–3, described in the main text, are the 10-, 20-, and 30-minute initial rest regressors, respectively. In the absence of an artifactual effect, the expected value of the root-mean-squared t-score across 100-parcels in 24 subjects would be 1.04, with a standard deviation of approximately 0.08.

T-test Summary Histograms

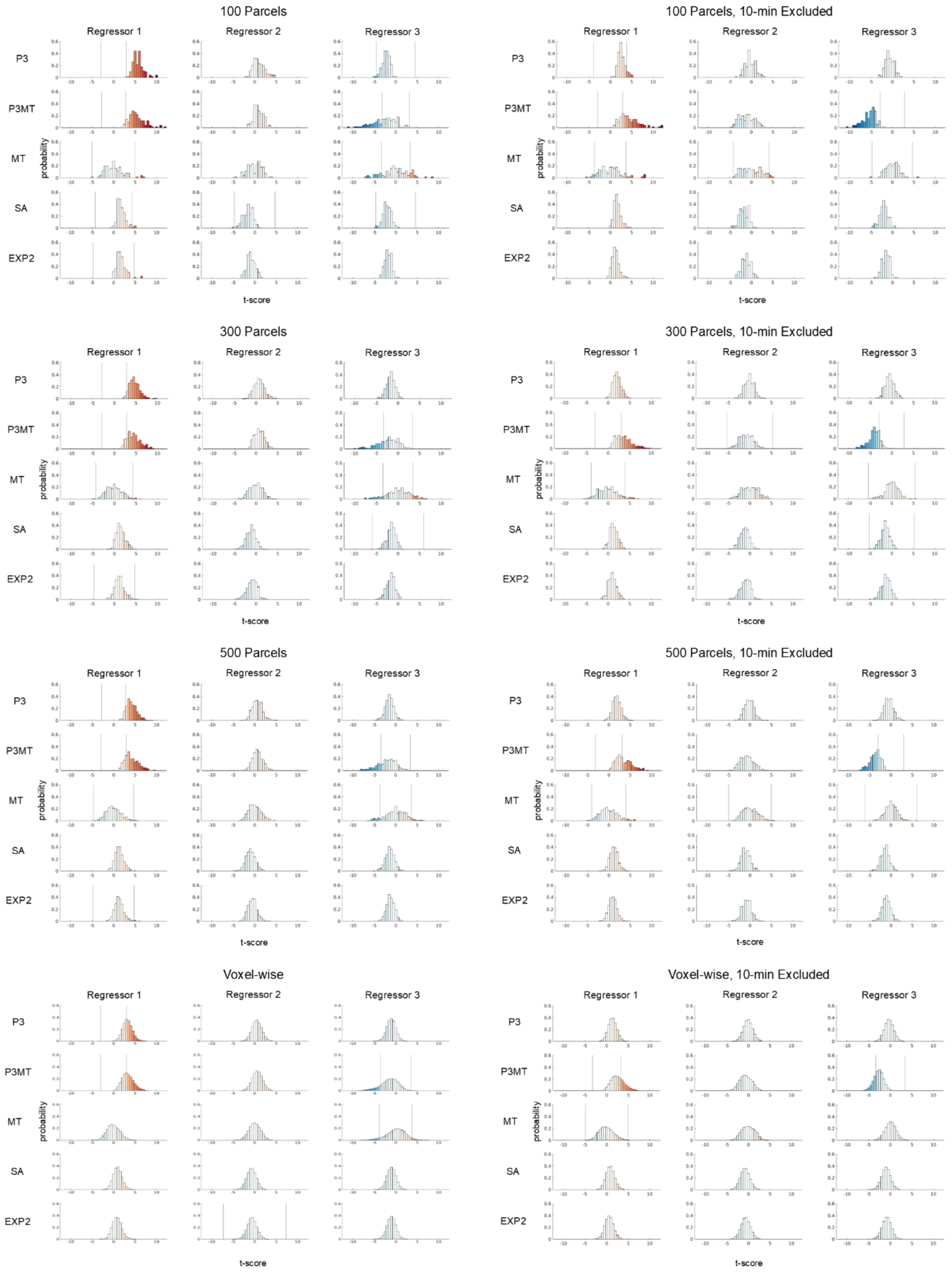

Figure S8: Histogram summaries of t-scores for each regressor / detrending method / spatial resolution combination for the constant-infusion resting-state data. Illustrative regressors and regression methods are identical as those used to generate results shown in Figs. 3 & 4; spatial resolutions are identical as those examined in the results shown in Fig. 5. To facilitate visualization of artifactual metabolic (de)activation, the color scale has a step-change at the ($p < 0.05$, FDR) significance point, if one exists; thus the color scale varies across sub plots.

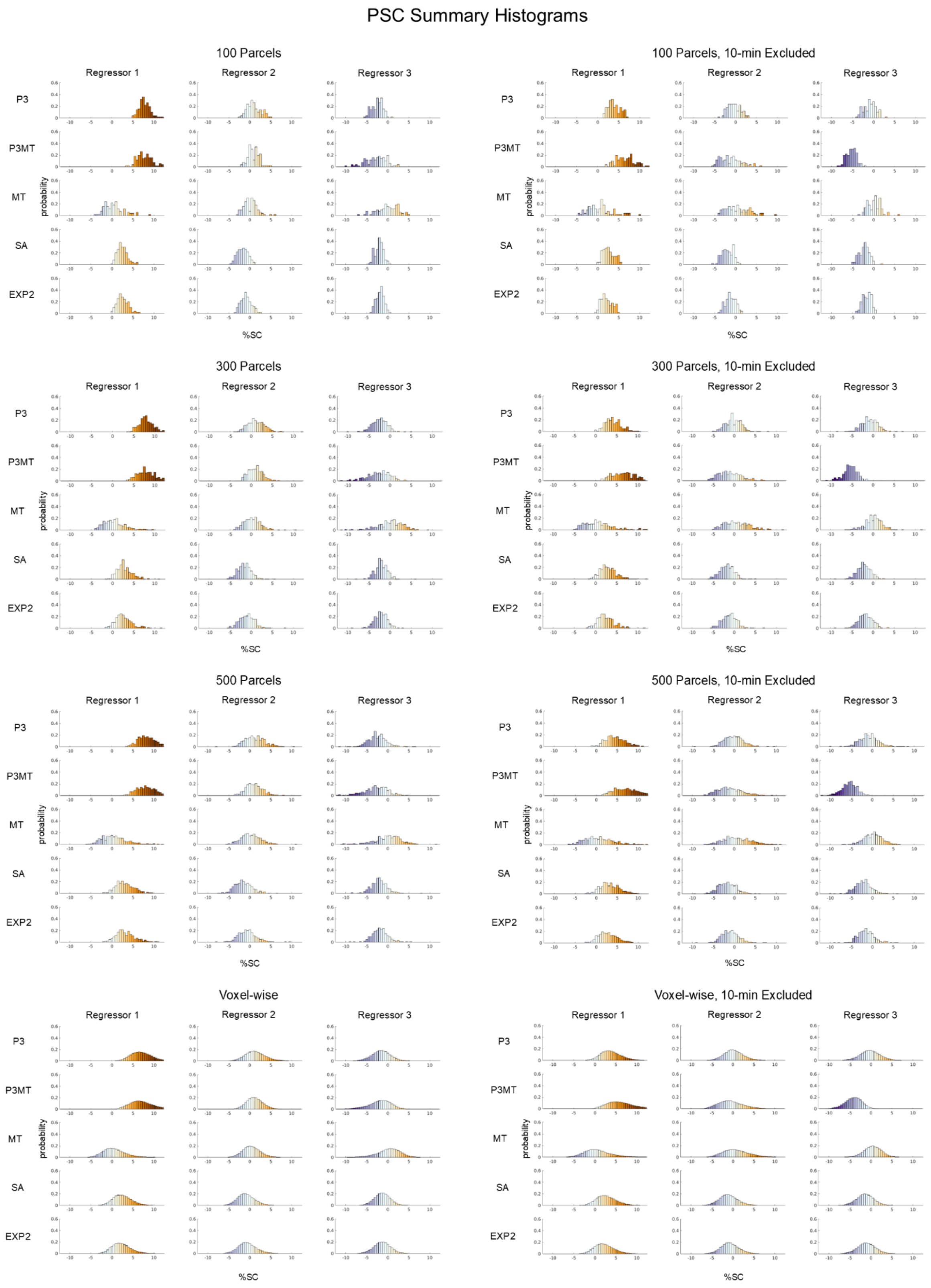

Figure S9: Histogram summaries of percent signal changes (PSCs) of the fPET TACs for each regressor / detrending method / spatial resolution combination for the constant-

infusion resting-state data. Illustrative regressors and regression methods are identical as those used in to generate results shown in Figs. 3 & 4; spatial resolutions are identical as those examined in the results shown in Fig. 5.
